# Bispecific antibodies targeting dual tumor-associated antigens in cancer therapy

**DOI:** 10.1007/s00432-020-03404-6

**Published:** 2020-09-28

**Authors:** Shuyu Huang, Sander M. J. van Duijnhoven, Alice J. A. M. Sijts, Andrea van Elsas

**Affiliations:** 1 Aduro Biotech Europe, Oss, The Netherlands; 2 Faculty of Veterinary Medicine, Department of Infectious Diseases and Immunology, Utrecht University, Utrecht, The Netherlands

**Keywords:** Bispecific antibodies, Dual targeting, Cancer therapy, Clinical trials, Literature review

## Abstract

**Purpose:**

Bispecific antibodies (BsAbs) have emerged as a leading drug class for cancer therapy and are becoming increasingly of interest for therapeutic applications. As of April 2020, over 123 BsAbs are under clinical evaluation for use in oncology (including the two marketed BsAbs Blinatumomab and Catumaxomab). The majority (82 of 123) of BsAbs under clinical evaluation can be categorized as bispecific immune cell engager whereas a second less well-discussed subclass of BsAbs targets two tumor-associated antigens (TAAs). In this review, we summarize the clinical development of dual TAAs targeting BsAbs and provide an overview of critical considerations when designing dual TAA targeting BsAbs.

**Methods:**

Herein the relevant literature and clinical trials published in English until April 1st 2020 were searched using PubMed and ClinicalTrials.gov database. BsAbs were considered to be active in clinic if their clinical trials were not terminated, withdrawn or completed before 2018 without reporting results. Data missed by searching ClinicalTrials.gov was manually curated.

**Results:**

Dual TAAs targeting BsAbs offer several advantages including increased tumor selectivity, potential to concurrently modulate two functional pathways in the tumor cell and may yield improved payload delivery.

**Conclusions:**

Dual TAAs targeting BsAbs represent a valuable class of biologics and early stage clinical studies have demonstrated promising anti-tumor efficacy in both hematologic malignancies and solid tumors.

## Introduction

The first therapeutic monoclonal antibody (mAb), muromonab-CD3 (OKT3), was approved by the Food and Drug Administration (FDA) more than 30 years ago, which marked the launch of a long mAb-based therapeutics campaign (Kung et al. 1979). Currently, antibody therapeutics represent the fastest growing class of drugs on the market with more than 70 antibody drugs approved and more than 550 in clinical study (Carter and Lazar 2018; Suurs et al. 2019). Within the large antibody-based therapeutic family, recently, bispecific antibodies have gained much interest in cancer therapeutic applications (Garber 2014). Compared to monospecific monoclonal antibodies, the potential advantages of BsAbs are listed here. By targeting two tumor-associated antigens (TAAs) that individually are not necessarily tumor-specific, in theory BsAbs achieve improved selectivity towards tumor, minimizing the side effects in normal tissues (Mazor et al. 2015, 2017). Since cancer is a complex and multifactorial disease, dual targeting could also be used to modulate two functional pathways in the tumor, thus avoiding resistance to the treatment (Lopez-Albaitero et al. 2017; Moores et al. 2016). Furthermore, BsAbs provided added functionality that cannot be achieved with a combination of two monospecific mAbs, such as redirecting specific immune cells to tumor cells (Zhukovsky et al. 2016), pre-targeting strategies (Boerman et al. 2003), half-life extension (Kontermann 2011) and delivery through the blood–brain barrier (Yu et al. 2011).

The first bispecific antibody, with the ability to bind to two different antigens at the same time, was generated by coupling rabbit antigen-binding fragments (Fabs) from two different polyclonal sera via mild re-oxidation 1960s (Nisonoff et al. 1960). At the time hope for this next generation, BsAb therapy were dampened due to manufacturing issues and clinical failure (Garber 2014). Over the past two decades, advances in biotechnology leading to improved protein engineering and manufacturing techniques have fueled the development of increasingly complex BsAbs with defined structure and biochemical, functional, and pharmacological properties (Brinkmann and Kontermann 2017). In oncology, two BsAbs have been approved for clinical treatment. Catumaxomab [CD3 × EpCAM (epithelial cell adhesion molecule)], was approved by the European Medicines Agency (EMA) in 2009 for the intraperitoneal treatment of malignant ascites although withdrawn in 2017 for commercial reasons. Blinatumomab (CD3 × CD19), was approved by the FDA in 2014 for the treatment of Philadelphia chromosome-negative B cell acute lymphoblastic leukemia (ALL) (Przepiorka et al. 2015; Seimetz et al. 2010). The approval of these two BsAbs has stimulated further attention and investment by pharmaceutical and biotech companies.

Bispecific antibodies are one of the rapidly growing new drug classes. With new BsAb clinical studies constantly emerging, keeping track is a challenging task. The various BsAbs including cell bridging, receptor inhibition/activation, co-factor mimicking and piggybacking BsAbs in oncology and autoimmune disease were summarized excellently in a recent review ( Labrijn et al. 2019). Therefore, we focus this review on the current state of the art of a less well-discussed subclass of BsAbs, targeting two tumor-associated antigens for oncology clinical development. We also discuss the factors that need to be carefully considered when designing BsAb targeting two TAAs and provide future perspectives for this field.

## Bispecific antibody formats

**Fig. 1:**
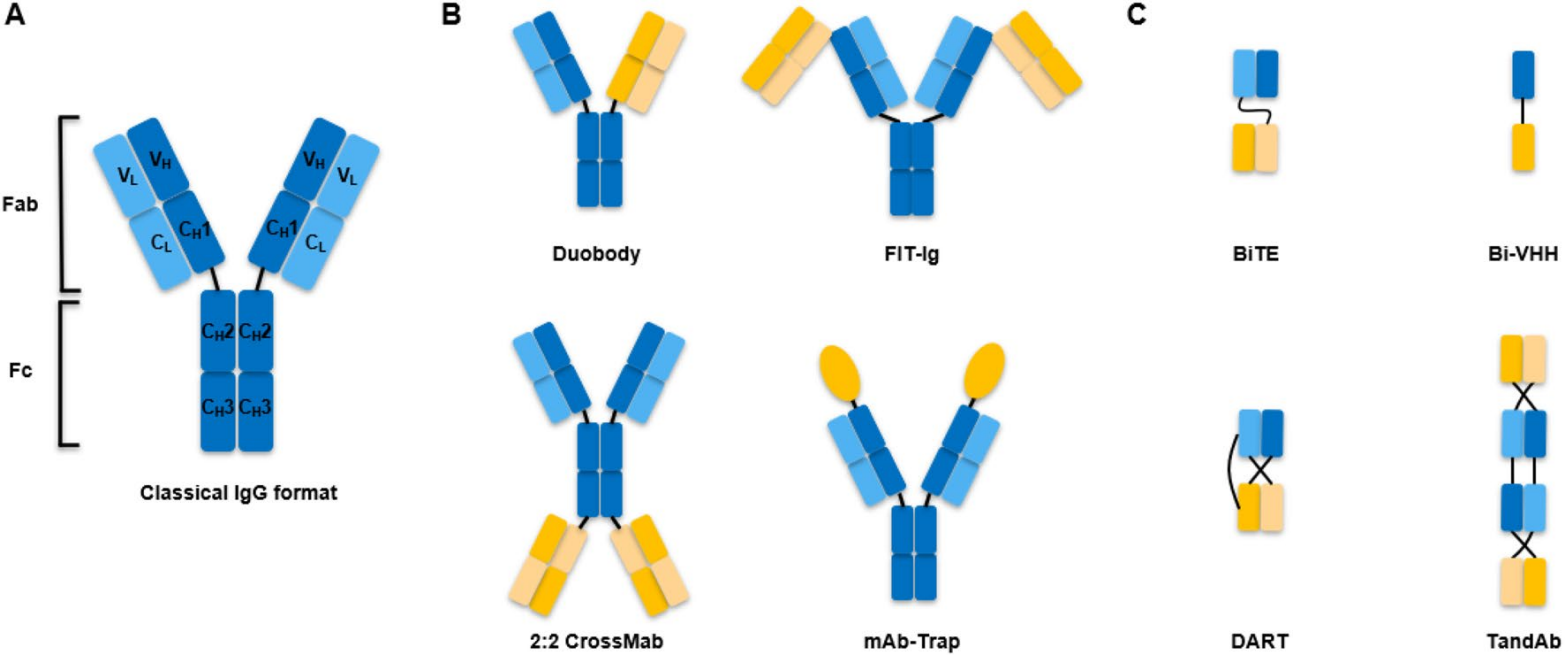
Schematic overview of the antibody structure and representations of several dual TAAs targeting BsAb formats with/without Fc tail. **a** The classical IgG structure; **b** representative Fc containing BsAb formats; **c** representative Fc less BsAb formats. *FIT-Ig* Fab-in-tandem immunoglobulin, *scFv* Single-chain variable fragment, *BiTE* Bispecific T cell engager, *VHH* variable domain of heavy chain, *DART* dual-affinity retargeting molecule, *TandAb* tandem diabody

Antibodies are grouped into five classes according to their constant region: IgG, IgM, IgA, IgD, and IgE. The basic structure of an IgG antibody is composed of two pairs of heavy-light chain polypeptide chains connected by interchain disulfide bonds and noncovalent bonds, resembling a “Y” shape complex, with a total molecular weight of ~ 150 kDa. An antibody can be also divided into functional parts: the antigen-binding fragments (Fab) and the fragment crystallizable (Fc) region (Fig. 1a). The Fc region is the tail region of an antibody that interacts with a receptor called the neonatal receptor, which is involved in regulating the IgG serum levels to prolong the antibody half-life. The Fc region also induces secondary immune functions that lead to immune-mediated target-cell killing, such as Antibody-dependent cell-mediated cytotoxicity (ADCC), Antibody-dependent cellular phagocytosis (ADCP) and Complement-dependent cytotoxicity (CDC) (Chiu and Gilliland 2016; Wang et al. 2018).

Typical antibodies are symmetric and monospecific, with two identical heavy-light chain polypeptide chains binding to the same epitope, while BsAbs are composed of two different antigen-binding regions. Hence, the formats of BsAb are much more complex and diverse than mAb. As a result of advances in protein and gene engineering, more than 100 different BsAb formats have been invented, with around one-fourth of those further developed into commercial platforms for bispecific antibody generation (Brinkmann and Kontermann 2017; Godar et al. 2018). The varied BsAb formats can be roughly divided into two classes depending on the presence of an Fc domain.

## Fc containing architecture

Fc region containing BsAbs mainly include Duobody( Labrijn et al. 2013), FIT-Ig (Gong et al. 2017), 2:2 CrossMab (Brunker et al. 2016), mAb-Trap (J. Yu et al. 2020) (Fig. 1b). Fc presence provides them with a relatively long in vivo half-life owing to its neonatal Fc receptor (FcRn)-mediated recycling processes (Roopenian and Akilesh 2007). In addition, the Fc region can also be designed to mediate secondary immune functions in accordance with the required mode-of-action (Table 1) (Scott et al. 2012). On the other hand, to address the chain association issues, protein engineering of Fc region containing BsAbs requires more effort which might compromise the physicochemical and biological characteristics or even affinity of the BsAb, eventually requiring additional analytical and quality testing (Klein et al. 2012).

**Table 1:** Comparison of Fc containing and Fc less bispecific antibodies

	Fc containing	Fc less
Representative platform	Duobody, CrossMab, FIT-Ig	BiTE, DART, TandAb
Representative drug	Catumaxomab	Bilncyto
Advantages	CMC: Good solubility and stability Curative effect: Induce secondary immune functions (ADCC, ADCP and CDC); long in vivo half-life	CMC: Small size, high yield, easy to produce Curative effect: Low immunogenicity; Fewer side-effects; Better tissue-penetrating capacity; For CD3 × antigen format, T cell mediated tumor cell killing is better than which Fc mediated
Disadvantages	Mis-paring and purification problems; relatively poor permeability of tumor tissue	Requires specific purification technology; require half-life extension or frequent dosing

*CMC* chemistry, manufacturing, and controls

## Fc less architecture

Fc-less BsAbs are composed of either single-chain variable fragment (scFv), variable domain of heavy chain of heavy-chain (VHH) or Fab fragment of two different antibodies, but without Fc region such as BiTE (Wolf et al. 2005), DART (Johnson et al. 2010), TandAb (Kipriyanov et al. 1999), Bi-VHH (Conrath et al. 2001), etc. (Fig. 1c). In the absence of an Fc region, these types of BsAbs are smaller in size and heavy-heavy chain mis-pairing issues are avoided, leading to relatively high yield, better tissue-penetrating capacity and less immunogenicity. But along with it came certain disadvantages such as the short in vivo half-life, decreased stability and a higher probability of aggregate formation (Table 1) (Ayyar et al. 2016; Kontermann and Brinkmann 2015; Velasquez et al. 2018).

## Dual TAAs targeting BsAbs

As of April 2020, over 123 BsAbs are under clinical evaluation in cancer patients (including marketed Blinatumomab and Catumaxomab). Among the 123 BsAbs, bispecific immune cell engagers (BICEs) are the dominant class of BsAbs (82 of 123), which target a receptor expressed on the immune cell surface with one arm and a tumor cell surface receptor with the other arm. Thus, they redirect specific immune effector cells to tumor cells. In this review, we focus on the dual TAAs targeting BsAbs.

**Fig. 2:**
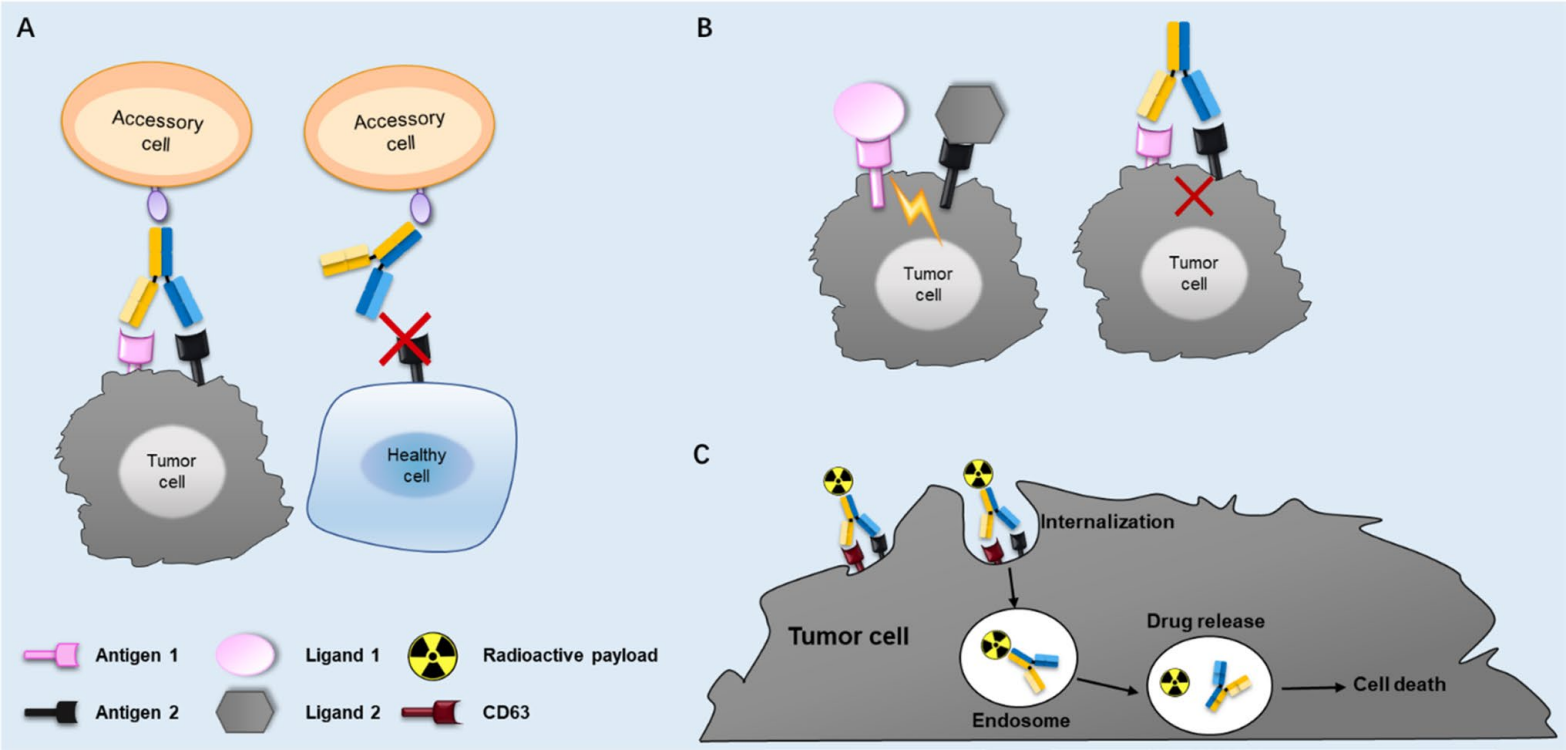
Proposed mechanisms of action (MOA) for dual TAAs targeting BsAbs. **a** Dual TAAs targeting BsAb binds to double antigen-positive cancer cells, but not single antigen-positive healthy cells; **b** dual signaling blockade; **c** enhanced payload delivery mediated by CD63 targeted BsAb

The strategy of dual TAAs targeting with a BsAb offers several advantages including increased tumor selectivity, modulation of two functional pathways in the tumor cell at the same time and improved payload delivery (Fig. 2). Although dual TAAs targeting BsAbs only represent a small portion of the 123 BsAbs undergoing clinical trials (9 of 123), the limited number of targets involved indicates its huge growth potential (Table 2).

**Table 2:** Clinical trials of dual tumor-associated antigens targeting bispecific antibodies

Antibody Name	Sponsor	Targets	Format	Diseases	Clinical studies
Zenocutuzumab, MCLA-128 PB4188	Merus	HER2 × HER3	Fab × Fab-Fc, IgG1, 1 + 1	Solid tumors harboring NRG1 fusion NSCLC harboring NRG1 fusion Pancreatic cancer Harboring NRG1 fusion	Phase II (NCT03321981)
OXS-1550, DT2219ARL	GT biopharma	CD19 × CD22	scFv × scFv, 1 + 1	Refractory B-lineage leukemia Relapsed B-lineage leukemia	Phase I/II (NCT02370160)
EMB01	EpimAb Biotherapeutics	EGFR × c-MET	Fab × Fab-Fc, IgG1, 2 + 2	Neoplasms Neoplasm metastasis Non-small-cell lung cancer	Phase I/II (NCT03797391)
JNJ-61186372	Janssen	EGFR × c-MET	Fab × Fab-Fc, IgG1, 1 + 1	Non-small-cell lung cancer	Phase I (NCT02609776 and NCT04077463)
TG-1801, NI-1701	TG Therapeutics	CD47 × CD19	Fab × Fab-Fc, IgG1, 1 + 1	B cell lymphoma	Phase I (NCT03804996)
IBI322	Innovent biologics	CD47 × PDL-1	Undisclosed	Advanced malignancies	Phase I (NCT04338659 and NCT04328831)
MCLA-158	Merus	EGFR × LGR5	Fab × Fab-Fc, IgG1, 1 + 1	Advanced/metastatic solid tumors Colorectal Cancer	Phase I (NCT03526835)
IMM0306	ImmuneOnco	CD20 × CD47	Fab × Ligand-Fc, IgG1, 2 + 2	Non-hodgkin lymphoma	Phase I (CTR20192612)
RO6874813 (RO7386)	Roche	DR5 × FAP	Fab × Fab-Fc, IgG1, 2 + 2	Advanced and/or metastatic solid tumors	Phase I (NCT02558140)

## Increased tumor selectivity

Many tumor-targeting monospecific mAbs not only eliminate tumor cells but also induce sometimes severe on-target toxicity towards healthy tissues. For example, anti-CD47 mAbs block a 'do not eat me' signal upregulated on tumor cells to evade macrophage-mediated phagocytosis but is also present on erythrocytes, platelets and other healthy cells. Anti-CD47 mAbs induce severe anemia and thrombocytopenia contributing to decision by Celgene to terminate the Phase I clinical study of CC-90002 (NCT02641002). To circumvent this, BsAbs were designed with a tumor-specific targeting arm to drive tumor-selective binding of an affinity optimized second arm targeting CD47. For instance, the TG-1801 (NI-1701), is a 1:1 IgG1 BsAb targeting CD19, a biomarker exclusively expressed on normal B cells and B-cell lineage malignancies, and CD47. BsAb TG-1801 could potentially overcome the limitation of CD47 monospecific targeting therapy by specifically blocking the 'do not eat me' signal only on B-cells. This is achieved by combining a low-affinity CD47 arm with a high-affinity CD19 arm, thereby reducing the risk of unwanted CD47 blockade in healthy cells (Buatois et al. 2018; Hatterer et al. 2019). Similarly, IMM0306, a CD20 x CD47 BsAb developed by ImmuneOnco has achieved remarkable therapeutic effects in various tumor models and showed no binding to human erythrocytes in pre-clinical study (Yu et al. 2020). Besides hematological malignancies, there are also BsAbs that work in a similar way to increase blockade/activation specificity in solid tumors, such as IBI322 and RO6874813 (RO7386). Whereas the depletion of healthy B cells can be tolerated to a certain degree in the treatment of B cell-derived tumors (e.g. by targeting CD19), this is not necessarily the case for targeting TAA expressed on solid tumors and associated healthy tissues. IBI322 is a CD47 × PDL-1 BsAb developed by Innovent Biologics which preferentially accumulated in PD-L1 positive solid tumors, thereby reducing the potential side effects due to the CD47 pathway blockade in healthy cells (Wang et al. 2020). In another example, RO6874813 is a 2:2 CrossMab that binds with high to fibroblast activation protein (FAP) on cancer-associated fibroblasts in tumor stroma and low affinity to death receptor 5 (DR5). The TNFR family member DR5 is often expressed on tumor cells and its activation induces apoptosis. FAP-driven binding enables docking of RO6874813 on cancer-associated fibroblasts increasing the local concentration of DR5 binding hyperclustering to potently induce apoptosis in tumor cells but not in normal cells (Brunker et al. 2016).

Strictly tumor-specific antigens useful for antibody targeting have yet to be identified in solid tumors. Although dual targeting of two tumor-selective antigens increases tumor selectivity over healthy cells expressing one antigen, it can be further improved. To address this, Mazor et al. generated different variants of EGFR × HER2 BsAbs each with, respectively, affinity optimized EGFR binding arms. Eventually, one EGFR × HER2 BsAb displayed much more preferential binding to EGFR-HER2 double-positive cells over EGFR single-positive cells (Mazor et al. 2017). Although the binding profile of this BsAb over HER2 single-positive cells was not reported, this study indicates that dual tumor-associated antigen targeting BsAb might require further tuning of binding affinity of one or both variable domains to achieve adequate tumor selectivity or specificity. In another example to achieve tumor-specific targeting, Banaszek et al. developed a Tri-specific T cell-engaging antibody derivative consists of two TAA targeting scFv and a CD3 binding fragment. Remarkably, this antibody comes in two complementary halves. Each half contains a TAA binding scFv fused to either the variable light (VL) or variable heavy (VH) chain domain of an anti-CD3 antibody. When the two complementary halves simultaneously bind their respective antigens on the same cell, they reconstitute the original CD3-binding site to engage T cells (Banaszek et al. 2019).

## Dual receptor signaling blockade

Cancer is a highly complex and multifactorial disease, involving multiple disease-driving proteins and cross-talking pathways. Cross-talk between different pathways supports a complex molecular network which may mediate tumor escape (Aleksakhina et al. 2019). Facilitated by inherent tumor heterogeneity, acquisition of drug resistance is often observed in patients who relapse after treatment with a single molecular targeted therapy.

Epidermal growth factor receptor (EGFR) is the first identified receptor tyrosine kinase, which plays essential roles in regulating cell proliferation, survival and differentiation. EGFR overexpression is associated with the development of epithelial malignancies, such as non-small cell lung cancer, ovarian cancer, colorectal cancer and prostate cancer (Nicholson et al. 2001). Tyrosine kinase inhibitors (TKIs) such as Gefitinib and Erlotinib that target the EGFR signaling cascade have been a clinical success over the past two decades, but also faced the challenge of drug resistance (Mok et al. 2009; Steins et al. 2018). For instance, in non–small cell lung cancer (NSCLC) patients demonstrated clinically meaningful response to first-generation EGFR tyrosine kinase inhibitors, but drug resistance was found to occur within a year or less (Kobayashi et al. 2005; Pérez-Soler et al. 2004). Although the second/third-generation TKIs demonstrates activity in drug-resistant patients, eventually they also develop acquired resistance to the TKIs due to new EGFR mutations (van der Wekken et al. 2016). Another important cause of drug resistance to TKIs is the activation of parallel RTK (Receptor Tyrosine Kinase) pathways. For instance, activation of Hepatocyte Growth Factor/Mesenchymal-Epithelial Transition factor (HGF/MET) pathway was shown to occur frequently bypassing EGFR TKI inhibitors (Bean et al. 2007; Engelman et al. 2007). With this in mind, two BsAbs (JNJ-61186372, Janssen; EMB01, EpimAb) targeting EGFR and c-MET were derived independently and are currently being tested in clinical studies. JNJ-61186372 is a humanized EGFR × c-MET BsAb generated using Fab arm exchange technology (Labrijn et al. 2013). JNJ-61186372 simultaneously blocks ligand-induced phosphorylation of EGFR and c-MET, and induces enhanced ADCC activity owning to the low-fucose-containing Fc carbohydrate. Moreover, JNJ-61186372 downregulated receptor expression on tumor cells thus preventing the drug resistance mediated by new emerging mutations of EGFR or c-MET (Castoldi et al. 2013; Moores et al. 2016). In a Phase I study (NCT02609776) which included 108 patients with advanced NSCLC, JNJ-61186372 has shown manageable safety profile and broad-spectrum anti-tumor efficacy in patients with EGFR exon 20 insertion, EGFR C797S mutation, MET amplification or resistance to Osimertinib, a third generation EGFR TKI (Park et al. 2020). Based on these data, FDA recently granted Breakthrough Therapy Designation (BTD) to JNJ-61186372 in NSCLC.

In another example, a HER2 × HER3 BsAb (Zenocutuzumab, also named MCLA-128, PB4188) is undergoing clinical evaluation for the treatment of patients with solid tumors harboring Neuroregulin1 (NRG1) fusion. NRG1 is a member of the EGF family that binds HER3 leading to the formation of a heterodimeric complex between HER2 and HER3. Patients treated for HER2 driven cancers are frequently found to escape from HER2 targeting agents via NRG1 activation of the HER3 pathway. NRG1 fusions represent actionable oncogenic driver mutations potentially useful to select patients most likely to respond to Zenocutuzumab. NRG1 fusions occur in ~ 3% NSCLC, ~ 1.5% pancreatic cancer and less than 1% of other cancers, and are detected frequently in KRAS–wildtype pancreatic ductal adenocarcinomas (PDAC) providing a potential drug target for those patients who do not benefit from KRAS inhibitors. Due to the high affinity to HER2, MCLA-128 docks on HER2 and blocks the formation of HER2/3 heterodimers and NRG1-fusion binding to HER3 simultaneously, thus inhibiting tumor cell proliferation (de Vries Schultink et al. 2020; Geuijen et al. 2018; Editorial 2019).

## Tumor delivery of toxic payloads

Antibody–drug conjugate (ADC) therapeutics combine the targeting precision of an antibody with the cytotoxic activity of a highly potent cytotoxic payload by conjugation to mAbs. Once the drug conjugated antibodies bind the antigens on tumor cell surface, ADCs are internalized by receptor-mediated endocytosis, and the toxic payload is released (Shim 2020). In the apparent absence of tumor-specific mAb targets or because tumor-selective targets not always internalize well, BsAbs may provide improved options compared to monospecific antibody-based ADC for tumor-selective delivery of highly potent chemical payloads.

For instance, the abundant clinical experience and approval of trastuzumab emtansine for the treatment of metastatic breast cancer confirmed that HER2 can be an effective ADC target. However, the internalization of HER2 targeted ADCs often relied on cross-linking of HER2 molecules while monomeric HER2 does not internalize well (de Goeij et al. 2016). To improve the internalization of HER2 targeted ADCs, a BsAb-based ADC targeting CD63 and HER2 was designed. CD63, also named lysosome-associated membrane glycoprotein 3 (LAMP3), is a member of the tetraspanin superfamily demonstrated to shuttle between the plasma membrane and intracellular compartments and is overexpressed in pancreatic cancer, gastric cancer and melanoma. The HER2 × CD63 BsAb showed strong internalization, lysosomal accumulation and cytotoxicity in HER2-positive tumor cells, and minimal internalization into HER2-negative cells (de Goeij et al. 2016).

CD19 and CD22 targeted therapy have been successful in the treatment of B cell lymphomas and rare Hairy Cell Leukemia (HCL), respectively, (Kochenderfer and Rosenberg 2013; Kreitman and Arons 2018). However, for CD19 targeted therapy, a sub-population of cancer cells in B-Lineage Leukemia patients turned to express CD22, thus escaped the killing mediated by CD19 targeted therapy (Fry et al. 2018). For CD22 targeted therapy, HCL represents only a small portion of patients with leukemia and expanding the use of the drug to a wider population of patients is critical. To overcome these resistance mechanisms, OXS-1550 (DT2219ARL), a CD19 × CD22 BsAb conjugated to a modified form of diphtheria toxin was developed and is currently being evaluated in Phase I study in patients with relapsed/refractory B cell lymphoma or leukemia (Bachanova et al. 2015; Schmohl et al. 2018).

Taken together, ADC-BsAbs can be designed to increase the selectivity of payload delivery, enhance its internalization or overcome the escape mechanisms of tumor cells, and may have huge potential as next-generation ADCs providing substantial advantage over monospecific antibody-based ADCs.

## Challenges and considerations for the development of dual TAAs targeting BsAbs

Abundant scientific rationale supports the development of BsAb for the treatment of multifactorial disease, such as cancer. BsAb have unique advantages compared to monospecific antibody, but there are also a number of specific challenges regarding bispecific antibodies development that need to be addressed (Li et al. 2020). In this respect, although the regulatory process for evaluation of monoclonal antibodies is well established, FDA published additional guidance for BsAb development programs in April 2019. The guidance for BsAb development programs highlighted additional consideration unique to BsAb development that address scientific rationale, chemistry, manufacturing, and controls (CMC), nonclinical pharmacology and clinical study. To support the development of a particular bispecific antibody, a strong scientific rationale should be provided including, but not limited to, adequate description of the two targets and the rationale for bispecific targeting [mechanism-of-action (MOA)], dose rationale and increased safety and/or efficacy as compared to similar monospecific products and available therapies. Diverse formats and engineering strategies enabling the design of BsAbs supporting a proposed MOA and the intended clinical application may also cause (1) unexpected attribute changes in BsAbs such as immunogenicity, antigen specificity, affinity and half-life or (2) production-related challenges including production yield, process-related impurities and stability (Atwell et al. 1997; Chailyan et al. 2011; Herold et al. 2017; Masuda et al. 2006). Different formats of BsAbs may require unique development considerations or technologies for each of them, but eventually, the BsAb products should be developed in accordance with standard monoclonal antibody development practices posing new challenges to CMC. Furthermore, during BsAb clinical studies, in addition to comparing the BsAb to the standard of care or placebo, in some cases, FDA may request a comparison of the BsAb to an approved monospecific product against the same antigen to inform the risk–benefit ratio. Based on the general indications provided in this FDA guidance, several critical factors need to be carefully considered when developing dual TAAs targeting BsAbs. These include (1) selection of target antigens, (2) affinity and biological effects of each arm, and (3) format utilized.

### Selection of target antigens

Rational target selection basically determines the MOA of BsAb and is the most important step for success. The preferred BsAb should enable novel biological function and therapeutic MOA which cannot be achieved using mAbs alone or in combination. Basic science supported a key role of c-MET in NSCLC patients developing resistance to EGFR TKIs, supporting design of JNJ-61186372 (EGFR × c-MET BsAb) and patient selection criteria leading to demonstrated anti-tumor activity in NSCLC patients with resistance to EGFR TKIs (Park et al. 2020; Yun et al. 2020). Interestingly, duligotuzumab (MEHD7945A), a BsAb targeting EGFR and HER3, showed no clinical benefit in comparison to cetuximab (anti-EGFR mAb) in phase 2 trials in patients with metastatic colorectal cancer or head and neck squamous cell carcinoma. Expression of HER3 determined by RNA or protein in tumor biopsies did not correlate with the response rate to duligotuzumab. Therefore, the researchers concluded that HER3 has a minor role in EGFR inhibitor naïve mCRC patients (Fayette et al. 2016; Hill et al. 2018). However, others believe that the disappointing results of the study were mainly due to improper selection of patients that were not resistant to prior cetuximab exposure (Saba 2017). Similarly, a phase III study (NCT02134015) of patritumab (HER3 inhibitor) in combination with erlotinib (EGFR inhibitor) for the treatment of NSCLC patients had failed before duligotuzumab (Liu et al. 2019; Yonesaka et al. 2017). Thus, the rationality of selecting EGFR and HER3 as targets for BsAb development requires further investigations. So far, BsAbs targeting dual TAAs have only involved a limited number of targets, with a main focus on ErbB family proteins. It will be interesting to assess BsAbs targeting novel target combinations developed for unmet clinical need.

### Affinity and biology effects of each arm

The affinity and biological activity of BsAb to each of the two antigens could have a critical impact on the final clinical outcome. Before JNJ-61186372, a BsAb against EGFR and c-MET (LY3164530) developed by Eli Lilly, did not enter phase II study due to toxicity and lack of data supporting a predictive biomarker. LY3164530 consisted of an IgG4 antibody targeting c-MET (emibetuzumab, LY2875358) and a single-chain variable fragment targeting EGFR (cetuximab) fused to the N-terminus of each heavy chain. By making use of these two existing antibodies (cetuximab and emibetuzumab), the affinity and activity for each individual arm in LY3164530 were fixed and the relative inhibition of EGFR versus c-MET and affinity to each individual antigen could not be adjusted to improve functionality. Significant toxicities of LY3164530 were recorded and found to be associated with EGFR inhibition but not c-MET inhibition, indicating that engineering the functionality of each arm might have improved its overall toxicity profile (Patnaik et al. 2018). In contrast, JNJ-61186372 was selected from a panel of EGFR × c-MET BsAbs based on functional activity and, similarly, zenocutuzumab came from an unbiased functional screening of aa panel of 545 BsAbs (Geuijen et al. 2018; Grugan et al. 2017). Moreover, a BsAb can sometimes exert a completely opposite activity compared to its two parental mAbs due to its format of conformation. For instance, a dual-variable-domain immunoglobulin (DVD-Ig) BsAb, generated by combining two well-validated antagonist anti-HER2 antibodies trastuzumab and pertuzumab, was shown to be a functional agonist of HER2 (Gu et al. 2014). Therefore, activity of a BsAb should not be assumed based on its parental mAbs, instead both affinity and biological activity should be investigated in an unbiased fashion following construction of the BsAb.

### Format utilized

The format of BsAb greatly influences its final physicochemical properties and biological functions. Over 100 different BsAb formats have been invented to solve many scientific or technical issues and their diversity enabled researchers to use them for various applications. A BsAb format suitable for all applications does not exist—the best format is the one that works well for desired application specifically (Brinkmann and Kontermann 2017). BsAbs proper designed with a well-chosen backbone can demonstrate enhanced anti-tumor efficacy and/or reduced side effects. Currently, the human IgG1 backbone is commonly used for dual TAAs targeting BsAbs mainly due to its well-known capacity to confer high exposure and long terminal half-life as well as inducing strong secondary immune functions. Many studies have demonstrated that small differences in the amino acid sequence of the CH2 and CH3 domain as well as the glycosylation profile of the Fc domain highly impact antibody thermal stability, pharmacokinetic properties and FcγR-mediated effector functions (Haraya et al. 2019; Kapelski et al. 2019; Regula et al. 2016; Roux et al. 1997; Zheng et al. 2011). The human FcγRIII, expressed on macrophages, monocytes, neutrophils, mast cells, and NK cells, binds antibodies with low glycosylation more tightly, thus inducing more potent ADCC effects (Satoh et al. 2006). For instance, ADCC of MCLA-128 was enhanced by low fucose glycoengineering using the GlymaxX® technology (ProBiogen) while retaining its binding to FcRn(de Vries Schultink et al. 2018); JNJ-61186372 was produced by a CHO cell line defective for protein fucosylation to enhance ADCC (Moores et al. 2016). However, in addition to differences in Fc region, variation in the variable region presentation and flexibility of the hinge region affect the functional activity of the IgG class. As reported by Kapelskia et al. the hinge region of human IgG subclasses showed different flexibility (IgG1 > IgG4 > IgG2, IgG1 being the most flexible) which significantly influenced the T cell redirection capacity of BsAb (Kapelski et al. 2019). Furthermore, in another example, eight anti-HER2 biparatopic BsAbs were generated from the same parental mAbs by DVD-Ig platform with different variable domain orientations or linker lengths. Interestingly, four BsAbs with same variable domain orientation showed strong agonistic activity while another four BsAbs with opposite orientation were antagonists. Further experiments demonstrated that the BsAb with a particular variable domain orientation could specifically prevent the heterodimer formation of EGFR/HER2 and HER2/HER3, thus forming more HER2 homodimers which lead to the activation of HER2 signal pathway (Gu et al. 2014). Unlike the factors influencing the potency of T cell engager antibodies which are well studied and reviewed (Ellerman 2019), the factors such as IgG subclass, variable domain orientation and length of hinge influencing BsAbs targeting dual TAAs are still largely unknown due to the completely different epitope topology, target geometry and distribution.

Compared to conventional monovalent BsAbs, more and more BsAb formats designed with multi-valence for each target have appeared and showed distinct advantages in particular cases. Cibisatamab (RG7802), a 2:1 CEA × CD3 BsAb, was optimized to have two CEA binding arms with low affinity individually but high avidity when combined, to increase the specificity to CEA^high^ tumor cells but spare CEA^low^ healthy cells. This setup facilitated Cibisatamab to bind to cells with > 10,000 CEA-binding sites/cell, which are most likely tumor cells (Bacac et al. 2016). For dual TAAs targeting BsAbs, the valence for each targeted TAAs should be considered individually based on specific properties of the targeted product profile. For instance, in the case of RO7386, a 2:2 BsAb targeting FAP and DR5 using high-affinity bivalent FAP arms ensured tumor-selective targeting, whereas bivalent low-affinity DR5 arms facilitated DR5 hyperclustering and killing of tumor cells (Brunker et al. 2016). Interestingly, for two BsAbs targeting EGFR and c-MET JNJ-61186372 used a 1:1 format while EMB01 used a 2:2 format. Early evidence supporting 1:1 design was that bivalent binding of c-MET invariably induced activation rather than inhibition due to dimerization (Wang et al. 2016). However, in pre-clinical studies, with bivalent binding to c-MET, EMB01 showed no c-MET activation in the absence of HGF. Furthermore, EMB01 achieved significant and sustainable tumor regression in the NCI-H1975-HGF CDX model, which was claimed to be more striking than the one achieved by JNJ-61186372 in a similar model. Such differences may be due to clustering induced by tetravalent antibody binding, which was demonstrated to enhance internalization and degradation for many receptors, including EGFR (Gong et al. 2017).

Besides IgG formats, the IgM format is also used for the development of BsAbs which by design provides more antigen binding sites than IgG format (Kaveri et al. 2012). For instance, IgM-2323 is a CD20 × CD3 bispecific IgM antibody developed by IGM Biosciences currently under clinical evaluation in Phase I for the treatment of patients with B cell Non-Hodgkin’s lymphoma (NHL) and other B cell malignancies (NCT04082936). In contrast to BsAb in other formats, IGM-2323 has 10 binding units to CD20 and one binding unit to CD3. Due to its 10 binding units for CD20, IGM-2323 is speculated to display very high avidity for CD20 expressing cancer cells including those with low CD20 expression that would escape from conventional anti-CD20 therapy (Keyt et al. 2020).

## Conclusions and prospects

Whereas, as a drug class monospecific mAbs have been established as a potent and credible option for cancer therapy, BsAbs are still in the exploration stage. Due to their unique design and structure, BsAbs bring unparalleled advantages compared to the monospecific mAbs, but also the challenges with respect to characterization and production. A challenge for the development of BsAbs is that each design and concept require unbiased analysis on a case-by-case basis. The different permutations and potential combinations of formats and targets makes every BsAb unique, requiring sound scientific exploration without drawing too many conclusions based on other experience. For the treatment of a multi-factorial disease, such as cancer, monospecific mAb-based therapy are always at risk of inducing drug resistance and tumor escape. Theoretically, BsAb based therapy could be a better solution and clinical data obtained so far supported this assumption, but much more is still needed. In this review, we have summarized the selection of target antigens, binding affinity, avidity and functional activity towards the two selected antigens as three critical factors to be considered in addition to the actual format for selection of clinical BsAb candidate drugs. BsAbs have huge potential to emerge as one of the most effective therapeutic biologicals and we firmly believe that BsAb-based therapies may revolutionize existing cancer treatment options in the future representing a big step forward in our fight against cancer.

## Abbreviations

ADC: Antibody drug conjugate
ADCC: Antibody-dependent cell-mediated cytotoxicity
ADCP: Antibody-dependent cellular phagocytosis
ALL: Acute lymphoblastic leukemia
BICEs: Bispecific immune cell engagers
BsAb: Bispecific antibody
BTD: Breakthrough therapy designation
CDC: Complement-dependent cytotoxicity
CMC: Chemistry: manufacturing: and controls
DR5: Death receptor 5
DVD-Ig: Dual-variable-domain immunoglobulin
EGFR: Epidermal growth factor receptor
EMA: European Medicines Agency
EpCAM: Epithelial cell adhesion molecule
Fab: Antigen binding fragment
FAP: Fibroblast activation protein
Fc: Fragment crystallizable
FcRn: Neonatal Fc receptor
FDA: Food and Drug Administration
HCL: Hairy cell leukemia
HGF: Hepatocyte Growth Factor
IgG: Immunoglobulin G
LAMP3: Lysosome-associated membrane glycoprotein 3
mAbs: Monoclonal antibodies
MET: Mesenchymal-epithelial transition factor
MOA: Mechanism of action
NRG1: Neuroregulin1
NSCLC: Non-small cell lung cancer
PDAC: Pancreatic ductal adenocarcinomas
RTK: Receptor tyrosine kinase
scFv: Single-chain variable fragment
TAAs: Tumor-associated antigens
TKIs: Tyrosine kinase inhibitors
VH: Variable heavy chain domain
VHH: Variable domain of heavy chain of heavy-chain
VL: Variable light chain domain
